# Knowledge, Beliefs, and Behaviors Regarding Colorectal Cancer Screening Among Koreans

**DOI:** 10.3390/healthcare14030344

**Published:** 2026-01-29

**Authors:** Shin-Young Lee

**Affiliations:** Department of Nursing, College of Medicine, Chosun University, Gwangju 61452, Republic of Korea; shinyoung0114@gmail.com

**Keywords:** colorectal cancer, knowledge, beliefs, screening, Koreans

## Abstract

**Background/Objectives**: Colorectal cancer (CRC) is a major health concern in South Korea, where incidence and mortality rates remain high despite the National Cancer Screening Program. Understanding the factors associated with CRC screening behaviors is essential for developing effective interventions. The purpose of this study was to examine the associations of sociodemographic characteristics, access to health care, knowledge, health beliefs, and cultural beliefs with CRC screening behaviors among Koreans. **Methods**: A cross-sectional survey was conducted with 648 Koreans aged 50 years and older at average risk for CRC. Participants completed questionnaires assessing sociodemographic characteristics, access to health care, knowledge, health beliefs, cultural beliefs, and CRC screening behaviors. Data were analyzed using descriptive statistics, bivariate logistic regression, and multivariate logistic regression with stepwise procedures. **Results**: Physician recommendation and perceived barriers were the strongest predictors of fecal occult blood test (FOBT) adherence, while physician recommendation, a usual source of health care, perceived benefits, and perceived barriers significantly predicted colonoscopy use. Perceived barriers reduced the likelihood of adhering to both, FOBT or colonoscopy (OR = 0.431, 95% CI = 0.316–0.588) or colonoscopy (OR = 0.432, 95% CI = 0.313–0.596), respectively, by 57%, whereas higher perceived benefits doubled the odds of colonoscopy participation (OR = 1.871, 95% CI = 1.331–2.631). Knowledge gaps were evident regarding CRC seriousness and the need for screening beginning at age 50 without symptoms. **Conclusions**: CRC screening participation among Koreans is associated primarily with access to care and health belief components. Encouraging physician recommendation and reducing perceived barriers are essential for improving screening rates. Culturally informed education and consideration of expanding colonoscopy services within the national cancer screening program is needed to further enhance CRC screening adherence.

## 1. Introduction

South Korea has one of the highest incidence rates of colorectal cancer (CRC) globally [1]. It ranked fifth and eleventh among 50 countries in age-standardized incidence rates of early-onset (diagnosed between ages 25 to 49 years) and late-onset (diagnosed between ages 50 to 74) CRC, respectively, based on cancer registry data from 1999–2017 [1]. Moreover, recent statistics from the National Cancer Information Center [2] report that CRC is the third most commonly diagnosed cancer among both Korean men and women in South Korea, with incidence rates of 78.2 and 46.3 per 100,000 population, respectively, in 2022. CRC is also the third leading cause of cancer-related deaths, accounting for 11.0% of all cancer deaths, with 9348 deaths reported in 2024 [3]. These data underscore the significant public health challenge posed by CRC in South Korea, highlighting the need for persistent actions to prevent, detect early, and treat the disease effectively.

To address the significant public health burden of CRC, the South Korean government has implemented national cancer screening programs, including the annual fecal occult blood test (FOBT) specifically for CRC screening [4]. These programs aim to facilitate early detection, improving treatment outcomes and reducing mortality associated with the disease. Individuals who receive a positive FOBT result through the national cancer screening program are advised to undergo a follow-up colonoscopy for more accurate and comprehensive assessment [4]. Colonoscopy plays a critical role in detecting abnormalities such as polyps or malignant lesions, ensuring timely intervention and appropriate treatment [5,6]. In adults aged 50 years and older, fecal occult blood testing yields positive results in approximately 2–7% of individuals [7,8]. When a fecal test yields a positive result, follow-up colonoscopy identifies advanced neoplasia—including precancerous colorectal lesions—in approximately 23% of cases [8].

According to Statistics Korea [9], consistent encouragement through the national cancer screening program increased CRC screening rates in Koreans aged 50 and older from 25.7% in 2012 to 41.6% in 2023. However, these rates still indicate that a substantial proportion of eligible individuals continue to avoid it. Furthermore, in a study involving 3464 adults aged 50 to 79, only 16.9% of the participants consistently adhered to CRC screening programs over a 12-year period, from 2007 to 2018 [10]. Low cancer screening rates contribute to delayed cancer detection, which, in turn, adversely affects survival outcomes [6,11,12,13]. For instance, a study reported that the annual up-to-date status for CRC screening (by FOBT, sigmoidoscopy, or colonoscopy) among individuals aged 51–71 years in the United States from 2000 to 2015 was significantly associated with a 25.5% reduction in CRC incidence and a 52.4% reduction in CRC mortality [12]. To address this public health concern, implementing targeted interventions to increase screening uptake is imperative. The development of effective, evidence-based intervention strategies to increase CRC screening utilization requires a comprehensive understanding of CRC screening behaviors, as well as the identification of key determinants that influence these behaviors among Koreans.

The literature review identified several significant predictors associated with participation in the FOBT screening, including physician recommendations [14,15,16,17,18,19], health beliefs such as perceived susceptibility [20], perceived severity [21], perceived benefit [18,20,22], perceived barriers [21,23,24], health temporal orientation (i.e., the perceived importance of detecting health problems early and being healthy in the future) [17], and fatalism [25]. Significant factors associated with colonoscopy uptake included physician recommendations [17,18,23], perceived severity [14], perceived barriers such as discomfort with the procedure [14,18,23], and knowledge [18,22,23,26,27]. Findings from an extensive literature review revealed several issues. First, although health beliefs are widely recognized as important correlates of CRC screening behaviors, limited empirical research has examined their role among Koreans, particularly in the context of culturally specific belief systems such as fatalism and crisis orientation (i.e., believing the health care system should be used as emergency response mainly). These culturally embedded beliefs may contribute to suboptimal CRC screening rates among Koreans; however, their influence remains underexplored. Second, existing CRC screening research in Koreans is limited by the lack of culturally validated belief measures, as instruments developed in Western culture, individualistic contexts may not accurately capture culturally embedded constructs. This mismatch can lead to construct bias and reduced interpretive validity. Therefore, CRC screening research should incorporate psychometrically sound and culturally appropriate belief scales to improve culturally validity and intervention effectiveness. The purpose of this study was to examine the associations of sociodemographic characteristics, access to health care, knowledge, health beliefs, and cultural beliefs with CRC screening behaviors, specifically FOBT and colonoscopy, among Koreans using psychometrically sound, culturally appropriate belief scales. The hypothesis that sociodemographic factors, access to health care, knowledge, health beliefs, and cultural beliefs are associated with CRC screening utilization among Koreans was tested in this study. These findings can provide empirical support for the development of effective interventions to increase CRC screening rates in Koreans.

## 2. Material and Methods

### 2.1. Study Design and Sample

This study was conducted using a paper-based questionnaire survey. The sample included Koreans who were born in South Korea, aged 50 and older, and at average risk of CRC—defined as having no history of Crohn’s disease, ulcerative colitis, CRC, or first-degree relative with CRC. A total of 648 Koreans participated in this study.

### 2.2. Data Collection

After obtaining approval from the Institutional Review Board of the affiliated university, participant recruitment began using a convenience sampling approach. The principal investigator and trained research assistants collaborated with community-based institutions to support recruitment efforts while maintaining voluntary participation. A total of 801 Koreans were recruited from churches and community centers in a metropolitan area of South Korea using a convenience sampling method between September 2022 and February 2023. Recruitment materials were distributed at these locations to invite eligible individuals who were willing and available to participate in this study. Koreans who were interested in participating in this study were asked to contact the principal investigator (PI). Individuals expressing interest in this study completed an eligibility assessment using a standardized screening tool approved by the university’s Institutional Review Board. Once participants were determined to be eligible, written informed consent was obtained after the PI provided a verbal explanation of this study’s objectives, procedures, potential risks, and confidentiality assurances. Participants were informed that they could withdraw from this study at any time. After obtaining the written consent forms, survey questionnaires were distributed in person to Koreans aged 50 and older. Those who met the inclusion criteria then filled out a self-administered, paper-based questionnaire, which required approximately 30 min to complete. A total of 648 individuals participated in this study (response rate = 80.9%). To protect anonymity, participants were assured that all data would be used solely for research purposes and that no personally identifiable information would be collected or shared.

### 2.3. Measures

Based on the health belief model [28], the cultural assessment model for health [29], and the Powe fatalism model [30], measures included sociodemographic characteristics (age, gender, marital status, education, employment, income, and health status), access to health care (usual source of health care—having a regular place or doctor to visit—and physician recommendation), knowledge of CRC and CRC screening, health beliefs related to CRC and CRC screening (susceptibility, severity, benefits, barriers, and self-efficacy), and cultural beliefs related to CRC (physical space, health temporal orientation, personal control, and fatalism), and CRC screening utilization (FOBT and colonoscopy). Knowledge of CRC and CRC screening was assessed among Koreans using items reflecting CRC incidence and mortality rates, as well as CRC screening guidelines. Health beliefs—including susceptibility (beliefs regarding the likelihood of developing a condition), severity (beliefs about seriousness of a condition and its consequences), benefits (beliefs in the effectiveness of the recommended action), barriers (beliefs about the costs or obstacles to taking the recommended action), and self-efficacy (confidence in one’s ability to act)—were measured using the Korean version of the health belief model scale [31]. The health belief model scale [31] demonstrated construct validity using exploratory and confirmatory factor analyses and showed good reliability, with Cronbach’s alpha coefficients ranging from 0.88 to 0.95 [31]. Cultural beliefs—including cancer fatalism (the belief that death is inevitable when cancer is present), health fatalism (notions of fate, luck, destiny, and predetermination regarding diseases or health conditions), health temporal orientation (perspectives on current health beliefs and behaviors in relation to future health concerns), and personal control (the perceived ability to plan activities to control or direct factors within the environment)—were measured using Korean versions of cultural belief scales [32]. Construct validity of the cultural belief scales was supported by exploratory and confirmatory factor analyses, and the scales showed good internal consistency (Cronbach’s α = 0.82–0.93) [32]. Health and cultural belief scales were measured on a five-point Likert scale ranging from strongly disagree to strongly agree [31,32]. Higher scores on each health belief scale denote more susceptibility to CRC (susceptibility scale), greater seriousness regarding CRC (severity scale), greater perceived benefits of FOBT (benefit scale), greater perceived barriers to FOBT (barrier scale), and greater self-efficacy regarding FOBT (self-efficacy scale). Minimum and maximum scores are 4 to 20 for susceptibility, 8 to 40 for severity, 5 to 25 for benefits, 9 to 45 for barriers, and 7 to 35 for self-efficacy. Higher scores on each cultural belief scale denote greater discomfort with the physical environment during the screening procedure (physical space scale), stronger preventive health or future orientation (health temporal orientation scale), greater internal and external control over early detection of health problems (personal control scale), and greater fatalism (fatalism scale). Minimum and maximum scores are 4 to 20 for physical space, 8 to 40 for health temporal orientation, 14 to 70 for personal control, and 15 to 75 for fatalism. Self-reported CRC screening utilization was assessed using the following questions: (1) whether the participant had ever undergone a CRC screening test (FOBT or colonoscopy) and (2) the date of the most recent CRC screening test.

### 2.4. Data Analysis

All statistical analyses were conducted using SPSS Version 30 [33]. After the completeness of the questionnaires was verified, the PI entered the data into the computer SPSS data file. The data were screened for data accuracy and missing data by the PI. All missing data were handled using multiple imputation of multivariate continuous data under the normal model before the analyses. Prior to model estimation, multicollinearity among the independent variables was assessed using variance inflation factors (VIFs). Model calibration and overall goodness of fit were evaluated using the Hosmer–Lemeshow test and calibration plots. Model discrimination was assessed by calculating the area under the receiver operating characteristic curve (AUC). Descriptive statistics were computed for participants’ sociodemographic characteristics, access to health care, knowledge, health beliefs, cultural beliefs, and CRC screening utilization (either FOBT or colonoscopy). We applied descriptive statistics, chi-square analyses, and independent *t*-tests to compare sociodemographic characteristics, access to health care, knowledge, health beliefs, cultural beliefs, and CRC screening utilization between community and religious centers. To examine the associations of the sociodemographic factors, access to health care, knowledge, health and cultural belief variables with FOBT and colonoscopy utilization among Koreans, the data analysis was performed in two steps. First, simple bivariate analyses were conducted to identify whether potential predictors were individually associated with CRC screening utilization. Second, multivariate analyses were performed to evaluate all potential predictors simultaneously. Variables that demonstrated significance in the bivariate analyses with a *p*-value < 0.05 were entered into a multivariate logistic regression model. Stepwise logistic regression using Wald statistics was then conducted. The stepwise Wald procedure was used for exploratory purposes to identify the most parsimonious set of predictors.

## 3. Results

### 3.1. Sample Characteristics

Table 1 presents the sociodemographic characteristics, access to health care, and CRC screening utilization of the participants. The mean age of the 648 Korean participants was 60.81 years. The majority of participants (86.4%) were married. More than half of the participants (60.8%) were women, and 46.9% reported having a usual source of health care. Additionally, 30.1% had received a physician recommendation for FOBT, and 41.5% had received a recommendation for colonoscopy. There was no statistically significant difference between participants recruited from community centers and religious centers in the sociodemographic characteristics, access to health care, knowledge, health beliefs, cultural beliefs, and CRC screening utilization, specifically FOBT or colonoscopy.

### 3.2. Knowledge, Beliefs Regarding CRC, and CRC Screening

Table 2 presents participants’ knowledge of CRC and CRC screening. More than 70% of participants answered most CRC and screening items correctly; however, two items scored low: CRC is a major cause of death in Koreans (correct responses of 56.9%); and CRC screening should start after age 50, unless there are any abnormal symptoms (correct responses of 54.5%). Table 3 summarizes means, standard deviations, and ranges of health and cultural beliefs, indicating higher perceived benefits and self-efficacy for colonoscopy than for FOBT.

### 3.3. Predictors of CRC Screening

Simple bivariate logistic regression analyses revealed that a gross household income of more than $40,000, having a usual source of health care, physician recommendation, knowledge, perceived benefits, perceived barriers, personal space, health temporal orientation, and external personal control were significantly associated with FOBT utilization in the previous year (Table 4). Similarly, bivariate logistic regression indicated that health status, having a usual source of health care, physician recommendation, knowledge, perceived benefits, perceived barriers, self-efficacy, personal space, health temporal orientation, and external personal control variables were significantly associated with colonoscopy in the previous 10 years (Table 4).

Table 5 presents the multivariable models for factors associated with CRC screening utilization. Physician recommendation and perceived barriers were statistically significant predictors of FOBT utilization in the previous year (*p* < 0.05) (Table 5). After controlling for other variables, Koreans who received a physician recommendation were twice as likely to have undergone FOBT (odds ratio (OR) = 2.079, 95% confidence interval (CI) = 1.373–3.150) compared with those who did not. With a one-unit increase in perceived barriers on the Likert scale, the likelihood of being adherent to FOBT decreased by 57% (OR = 0.431, 95% CI = 0.316–0.588). The odds of colonoscopy utilization in the previous 10 years were six times higher among respondents who received a physician recommendation than among those who did not (OR = 6.073, 95% CI = 3.749–9.837). Additionally, Koreans with a usual source of health care were 1.5 times more likely to have had an FOBT (OR = 1.510, 95% CI = 1.000–2.278), with a *p*-value of 0.05. Koreans who perceived greater benefits were nearly twice as likely to have undergone colonoscopy (OR = 1.871, 95% CI = 1.331–2.631). With a one-unit increase in perceived barriers, the likelihood of being adherent to colonoscopy decreased by 57% (OR = 0.432, 95% CI = 0.313–0.596).

## 4. Discussion

This study examined sociodemographic characteristics, access to health care, knowledge, health and cultural beliefs, and FOBT and colonoscopy utilization for CRC screening among Koreans aged 50 and older. Among these variables, the factors most strongly associated with CRC screening were access to health care (physician recommendation and having a usual source of health care) and health beliefs (perceived benefits and perceived barriers). More specifically, the findings indicate that, among all examined predictors, only physician recommendation and perceived barriers were significantly associated with FOBT utilization in the previous year in Koreans. Having a usual source of health care, physician recommendation, and perceived benefits, as well as perceived barriers, were significantly associated with colonoscopy utilization in the previous 10 years. These results suggest that, with regard to access to health care, individuals should be encouraged to visit health care providers regularly and to receive physician recommendations for CRC screening. In terms of health beliefs, the findings highlight the importance of implementing targeted interventions to enhance perceptions of the benefits of CRC screening while reducing perceived barriers.

Among all variables in this study, physician recommendation was the single most important factor affecting both FOBT (OR = 2.079) and colonoscopy (OR = 6.073) in Koreans. Physician recommendation consistently emerged as the most powerful determinant of CRC screening participation across diverse populations, including Koreans, Chinese, Asian Americans, and other ethnic groups [15,17,18,19,23,26,34,35,36,37]. Physician recommendation consistently served as the most influential facilitator of CRC screening, outweighing other social or cultural factors [17,18,19,20,22,23,26,34,38,39]. Targeted physician engagement interventions—such as personalized recommendation letters, phone calls, and educational mailers—were shown to markedly increase screening rates [40,41,42], highlighting that enhancing provider communication remains a key strategy to improve CRC screening participation. Notably, Korean physicians predominantly recommended colonoscopy (80%) [35], and their strong preference for colonoscopy over FOBT significantly influenced the national cancer screening trends, sustaining colonoscopy uptake despite free FOBT programs [15,35]. Collectively, these findings underscore the need for provider-centered strategies to enhance CRC screening adherence.

Among the health belief constructs, perceived benefits and perceived barriers were significantly associated with CRC screening adherence, which is consistent with previous literature [18,20,22,24,43]. Specifically, a one-unit increase in perceived barriers on the Likert scale was associated with a 57% decrease in the likelihood of adherence to both FOBT (OR = 0.431, 95% CI = 0.316–0.588) and colonoscopy (OR = 0.432, 95% CI = 0.313–0.596). Conversely, individuals with higher perceived benefits were nearly twice as likely to have undergone colonoscopy (OR = 1.871, 95% CI = 1.331–2.631). Unexpectedly, perceived benefits of FOBT were not significantly associated with actual utilization, suggesting that participation in FOBT is primarily driven by its institutional requirement within the National Cancer Screening Program. Despite this structural facilitation, Korean adults tend to have limited confidence in the reliability of FOBT for cancer detection. In this study, 70.5% of participants correctly identified that “a fecal blood test is a diagnostic test for colon cancer,” whereas 78.5% correctly recognized that “colonoscopy is a diagnostic test for colon cancer.” Furthermore, the mean perceived benefit scores for FOBT and colonoscopy were 3.86 and 3.96, respectively. Although the participants acknowledged the potential advantages of FOBT, there remains a prevailing belief that colonoscopy offers superior diagnostic efficacy.

Although cultural beliefs (e.g., physical space, health temporal orientation, personal space, and fatalism) did not reach statistical significance, the instrument used to measure health beliefs [31] was culturally adapted, and several culturally relevant items were embedded within the health belief constructs. For example, familism is an important cultural value for Koreans [16,25,44,45,46,47], and items reflecting this value were incorporated into both the perceived benefits and perceived barriers subscales [31]. In the benefits subscale, items included: “Colonoscopy can enable me to reduce a burden to my family by finding colon cancer early” and “Colonoscopy can enable me to take care of my family by finding colon cancer early.” In the barriers scale, items included: “I tend to put off having a stool blood test due to my family obligation” *and* “Fear of being a burden to the family, if diagnosed with colon cancer, would keep me from having a stool blood test.” Familism—the cultural value of placing the interests and well-being of the family before those of the individual—plays a dual role in cancer screening decisions [16,25,31,45]. It may function as a significant barrier: Koreans may fear that a cancer diagnosis would burden their family, leading them to avoid screening to prevent potential financial and emotional strain on their loved ones [17]. On the other hand, familism can serve as a strong motivator. The desire to remain healthy for the family—and to continue providing care and support—can be a compelling reason to participate in preventive screening [16].

Within the knowledge scale, two items demonstrated relatively low levels of accuracy. Specifically, 56.9% of participants correctly identified the statement “Colon cancer is a major cause of death among Koreans,” while 54.5% correctly responded to the statement “Colorectal cancer screening should begin after age 50, unless abnormal symptoms are present.” The former item assesses knowledge regarding the seriousness of CRC, whereas the latter measures preventive orientation. A predominant belief observed in both Koreans and Korean Americans is crisis health orientation, which posits that one should see a doctor only when symptoms are present [25,32]. Under this orientation, being symptom-free is often equated with being healthy, a perspective that directly undermines the core principle of preventive screening for asymptomatic conditions. This orientation contributes to delays in seeking care until a disease is potentially more advanced. Reflecting this crisis health orientation, the presence of physical symptoms remains a primary driver for seeking medical evaluation, whereas action is often deferred until a health problem becomes apparent. Therefore, interventions that strengthen of the seriousness of colorectal cancer and promote a stronger preventive orientation are needed to enhance screening participation.

Although FOBT is offered free of charge through the national cancer screening program, the screening rate in this study was low at 19.8%, whereas 71% of participants reported having undergone colonoscopy. In the knowledge items, 70.5% correctly identified that “A fecal blood test is a screening test for colon cancer”, and 78.5% correctly answered that “Colonoscopy is a diagnostic test for colon cancer.” This indicated that most participants were aware that both FOBT and colonoscopy serve as diagnostic tests for CRC. Among the barrier items, more than 30% of participants agreed with the statement, “Other colon cancer screening options such as colonoscopy are more accurate than a stool blood test, which would keep me from having a stool blood test”. This finding suggests that many individuals prefer colonoscopy over FOBT due to perceptions of greater diagnostic accuracy. Therefore, to increase CRC screening rates among Koreans, interventions should simultaneously address economic and psychosocial factors. Moreover, within the National Cancer Screening Program, it may be necessary to reconsider expanding access to colonoscopy rather than relying primarily on FOBT.

This study has several limitations. First, FOBT and colonoscopy were the only CRC screening modalities measured as outcome variables; other CRC screening options, such as sigmoidoscopy, were not assessed in this study. Because individuals may choose sigmoidoscopy, including additional CRC screening options in future analyses may provide a more comprehensive understanding of CRC screening behaviors. Second, the generalizability of the findings may be limited due to the use of voluntary convenience sampling. Recruitment via community centers and religious organizations may overrepresents socially connected individuals. Third, key variables were measured using self-reported data, which may be subject to self-report bias. In particular, information regarding dates of events relied on participants’ recall and may therefore be affected by recall bias, potentially leading to misclassification or measurement error. Such inaccuracies could have influenced the observed associations and should be considered when interpreting the findings. Fourth, this study is subject to potential residual confounding. Although the analyses adjusted for several known and theoretically relevant covariates, unmeasured or imperfectly measured confounding factors may still remain. Lastly, a limitation is the use of stepwise modeling, which is data-driven and may yield unstable estimates and an overfitted model, potentially limiting reproducibility and generalizability.

## 5. Conclusions

This study underscores that access to health care—particularly physician recommendation—and salient health beliefs such as perceived benefits and barriers are pivotal determinants of CRC screening behavior among Koreans aged 50 and older. Despite the implementation of the national cancer screening program, there remains a lack of knowledge regarding the seriousness of CRC and the need for adults aged 50 and older to begin screening even in the absence of symptoms, indicating the need for enhanced awareness and education. These findings call for provider-driven communication strategies, culturally informed educational interventions, and consideration of expanding colonoscopy services within the national cancer screening program to enhance CRC screening adherence.

## Figures and Tables

**Table 1:** Sociodemographic and health-related characteristics of Koreans (N = 648).

Variable	n (%)	M ± SD	Range
**Sociodemographic Characteristics**			
Age (year)			
50–64	430 (66.4)	60.81 ± 7.80	50–85
≥65	218 (33.6)		
Gender			
Male	254 (39.2)		
Female	394 (60.8)		
Marital status			
Currently married	560 (86.4)		
Not married	88 (13.6)		
Education			
≤High school graduate	357 (55.1)		
>High school graduate	291 (44.9)		
Employment			
Employed	334 (51.6)		
Unemployed	314 (48.4)		
Household income			
≤$40,000	385 (59.7)		
>$40,000	263 (40.3)		
Health status			
Good	264 (40.7)		
Fair	325 (50.2)		
Poor	59 (9.1)		
**Access to health care**			
Usual source of health care			
Yes	304 (46.9)		
No	344 (53.1)		
Having physician recommendation for FOBT			
Yes	195 (30.1)		
No	453 (69.9)		
Having physician recommendation for colonoscopy			
Yes	269 (41.5)		
No	379(58.5)		
**Colorectal cancer screening utilization**			
FOBT in the previous year			
Yes	128 (19.8)		
No	520 (80.2)		
Colonoscopy in the previous 10 years			
Yes	460 (71.0)		
No	188 (29.0)		

Note. FOBT = fecal occult blood test.

**Table 2:** CRC and screening knowledge items: correct responses (N = 648).

Knowledge of CRC and Screening Item	N (%)
Colon cancer is the third most commonly diagnosed cancer in Korea (T)	509 (78.5)
Colon cancer is a major cause of death in Koreans (T)	369 (56.9)
The risk factors for colon cancer are large intake of red meat, obesity, alcohol consumption, and genetic factors (T)	568 (87.7)
Colon cancer begins with a lump that grows in the large intestine (T)	547 (84.4)
When a lump is found in colon cancer, it is mostly cancer (F)	458 (70.7)
The main symptoms of colon cancer are diarrhea, constipation, bloody stools, fatigue, and indigestion (T)	539 (83.2)
Colorectal cancer screening should start after age 50, unless there are any abnormal symptoms (T)	353 (54.5)
There is no way to prevent colon cancer (F)	519 (80.1)
Fecal blood test is a diagnostic test for colon cancer (T)	457 (70.5)
Colonoscopy is a diagnostic test for colon cancer (T)	509 (78.5)
M ± SD	7.45 ± 1.63

Note. Values are N (%). (T) = correct response is true. (F) = correct response is false.

**Table 3:** Health and cultural beliefs (n = 648).

Beliefs	M ± SD	Range
**Health Beliefs**		
Susceptibility	2.29 ± 0.03	1–5
Severity	2.96 ± 0.03	1–5
Benefits (FOBT)	3.86 ± 0.02	1–5
Benefits (Colonoscopy)	3.96 ± 0.02	1.33–5
Barriers (FOBT)	2.46 ± 0.03	1–4.82
Barriers (Colonoscopy)	2.28 ± 0.26	1–4.82
Self-efficacy (FOBT)	3.60 ± 0.03	1–5
Self-efficacy (Colonoscopy)	3.88 ± 0.21	1.19–5
**Cultural Beliefs**		
Personal space (FOBT)	2.69 ± 0.04	1–5
Personal space (Colonoscopy)	2.80 ± 0.03	1–4.81
Health temporal orientation	3.51 ± 0.02	2.38–5
External personal control	2.13 ± 0.02	1–5
Internal personal control	3.73 ± 0.03	1.5–5
Health fatalism	2.68 ± 0.26	1–5
Colorectal cancer fatalism	2.22 ± 0.25	1–4.87

**Table 4:** Factors associated with fecal occult blood test and colonoscopy utilization from bivariate logistic regression (N = 648).

Factors	FOBT in the Previous Year				Colonoscopy in the Previous 10 Years			
	B	SE	OR (95% CI)	*p*-Value	B	SE	OR (95% CI)	*p*-Value
**Sociodemographic Characteristics**								
Age (year) (ref. 50–64)								
≥65	0.184	0.168	1.202 (0.865–1.671)	0.273	−0.092	0.182	0.912 (0.638–1.304)	0.614
Gender (ref. Male)								
Female	0.244	0.162	1.276 (0.930–1.752)	0.131	−0.181	0.179	0.835 (0.588–1.186)	0.313
Marital Status (ref. No)								
Currently married	−0.181	0.233	0.834 (0.528–1.316)	0.436	0.392	0.241	1.480 (0.923–2.372)	0.104
Education								
>High school graduate	−0.120	0.159	0.887 (0.650–1.210)	0.449	0.257	0.176	1.293 (0.917–1.824)	0.143
Employment								
Employed (ref. No)	−0.188	0.158	0.828 (0.607–1.130)	0.234	−0.183	0.174	0.832 (0.592–1.170)	0.291
Household income (ref. ≤ $40,000)								
>$40,000	−0.441	0.162	**0.644** (0.469–0.884)	**0.006**	0.266	0.179	1.304 (0.918–1.854)	0.139
Health status (ref. Poor)				0.24				**0.003**
Fair	0.387	0.406	1.473 (0.665–3.264)	0.34	0.362	0.29	1.437 (0.813–2.538)	0.212
Good	0.607	0.408	1.835 (0.825–4.082)	0.137	0.868	0.303	2.383 (1.315–4.318)	**0.004**
**Access to health care**								
Usual source of health care (ref. No)	0.817	0.162	**2.264** (1.647–3.111)	**<0.001**	1.014	0.185	**2.757** (1.919–3.962)	**<0.001**
Having a doctor’s recommendation (ref. No)	1.568	0.201	**4.796** (3.234–7.111)	**<0.001**	1.997	0.234	**7.365** (4.654–11.656)	**<0.001**
**Knowledge**	0.103	0.049	**1.108** (1.007–1.219)	**0.035**	0.152	0.052	**1.164** (1.052–1.289)	**0.003**
**Health beliefs**								
Susceptibility	−0.195	0.11	0.823 (0.662–1.022)	0.077	−0.136	0.12	0.873 (0.690–1.105)	0.258
Severity	−0.122	0.095	0.885 (0.735–1.065)	0.197	−0.088	0.104	0.916 (0.747–1.122)	0.395
Benefits	0.585	0.14	1.794 (1.364–2.361)	**<0.001**	0.715	0.155	**2.044** (1.508–2.770)	**<0.001**
Barriers	−1.124	0.138	0.325 (0.248–0.426)	**<0.001**	−1.009	0.15	**0.364** (0.272–0.489)	**<0.001**
Self-efficacy	0.047	0.106	1.048 (0.852–1.290)	0.656	0.582	0.163	**1.789** (1.301–2.461)	**<0.001**
**Cultural Beliefs**								
Personal space	−0.344	0.089	**0.709** (0.596–0.844)	**<0.001**	−0.598	0.123	**0.550** (0.432–0.699)	**<0.001**
Health temporal orientation	0.607	0.166	**1.834** (1.324–2.541)	**<0.001**	0.849	0.182	**2.338** (1.636–3.342)	**<0.001**
External personal control	−0.291	0.132	**0.748** (0.577–0.969)	**0.028**	−0.498	0.142	**0.609** (0.461–0.804)	**<0.001**
Internal personal control	0.092	0.108	1.097 (0.888–1.355)	0.391	0.213	0.117	1.237 (0.984–1.554)	0.068
Health fatalism	−0.162	0.122	0.850 (0.670–1.079)	0.182	−0.214	0.133	0.807 (0.622–1.047)	0.107

Note. Ref. = reference.

**Table 5:** Factors significantly associated with fecal occult blood test and colonoscopy utilization from multivariate logistic regression (N = 648).

	B	SE	OR (95% CI)	*p*-Value
**FOBT** in the Previous Year				
Physician recommendation (ref. No)	0.732	0.212	2.079 (1.373–3.150)	<0.001
Barriers	−0.842	0.158	0.431 (0.316–0.588)	<0.001
Chi-square likelihood ratio test = 50.980, df = 2, *p*-value < 0.001				
Colonoscopy in the previous 10 years				
Usual source of health care (ref. No)	0.412	0.210	1.510 (1.000–2.278)	0.050
Physician recommendation (ref. No)	1.804	0.246	6.073 (3.749–9.837)	<0.001
Benefits	0.627	0.174	1.871 (1.331–2.631)	<0.001
Barriers	−0.839	0.164	0.432 (0.313–0.596)	<0.001
Chi-square likelihood ratio test = 152.158, df = 4, *p*-value < 0.001				

Note. Ref. = reference.

## Data Availability

All the data are available from the corresponding author upon reasonable request due to privacy restrictions. The data are not publicly available due to [privacy restrictions].
